# An analysis of flavor descriptors on tobacco products in the Philippines: Regulatory implications and lessons for low- and middle-income countries

**DOI:** 10.1186/s12992-024-01072-6

**Published:** 2024-09-09

**Authors:** Samantha J. Ackary, Patrik James DL. Cabrera, Alen Josef A. Santiago, Gianna Gayle H. Amul

**Affiliations:** School of Government, Ateneo de Manila University, Quezon City, Philippines

**Keywords:** Tobacco, Flavor, Packaging, Philippines, Policy

## Abstract

**Background:**

Historically, tobacco companies have used flavored tobacco products to enhance the appeal of tobacco consumption, encourage initiation and experimentation of tobacco use, and contribute to sustained tobacco use. While flavored tobacco products are regulated in several countries, there is no existing regulation on flavored tobacco products in the Philippines, specifically for cigarettes and cigars. This study aims to update evidence on the flavored tobacco product landscape in the Philippines by assessing both the flavor descriptors and flavor imagery featured on cigarette and cigar packaging.

**Results:**

We collected 106 cigarette and cigar products from four major cities in the National Capital Region, Balanced Luzon, Visayas, and Mindanao. Of these 106 cigarette and cigar products, 62 (58.49%) had flavor descriptors. Three crushable capsule products did not feature any flavor descriptor but were included for flavor imagery examination. We identified five categories of flavor descriptors: menthol, concept descriptors, tobacco, beverages, and other flavors. Out of 62 packs, ten featured more than one flavor descriptor on the packaging. Menthol flavor descriptors comprised the majority of flavor descriptors. Imagery and other graphic elements closely resonate with and enhance the flavor descriptors found on these packs.

**Conclusions:**

This study aimed to update the evidence on the flavored tobacco product landscape in the Philippines and address their absence of regulation. Regulating flavored tobacco products requires a comprehensive policy approach complemented by complete enforcement. Flavor substances, flavor descriptors, and flavor imagery must be regulated altogether; however, it is ideal to enforce a ban on flavored tobacco products in compliance with the WHO FCTC, to which the Philippines is a signatory. Policymakers should consider plain packaging as an intervention to eliminate the appeals associated with flavored tobacco products.

## Background

Historically, the Philippines has grappled with high records of smoking prevalence. According to the 2021 Global Adult Tobacco Survey, 14.4 million adults aged 15 and above currently smoke tobacco, and 17.4 million adults smoke manufactured cigarette products [1]. The 2019 Global Youth Tobacco Survey found that 10.8% of students currently smoke tobacco, while 10% of youth aged 13–15 currently smoke cigarettes [2]. As a result, the country has experienced significant health and economic burdens linked to tobacco use. Every year, 112,000 Filipinos die from tobacco-related diseases [3] in which the smoking-attributable death rate stands at 200 per 100,000 population [4]. Meanwhile, the economic costs linked to smoking in the Philippines are estimated at over Php 269.3 billion (approximately USD 4.7 billion), where smoking-related health expenditure accounts for Php 23.1 billion (approximately USD 412 million), or roughly 4.9% of the nation’s total health expenditure [5]. The total economic cost of smoking drains 2.5% of the country’s GDP [4]. In 2024, the gradual tobacco tax increases are predicted to result in a projected revenue of U.S. $8.9 billion [6]. So far, the tobacco taxation increases led to a decrease in smoking from 23.5% in 2015 to 17.7% in 2021 [6].

The tobacco industry in the Philippines was once described as “the strongest tobacco lobby in Asia” [7] due to its aggressive strategies to boost sales, advance market share, and increase its customer base. One of several growing tactics to boost tobacco consumption is through flavored tobacco products. Flavored tobacco products are tobacco products infused with flavorings such as menthol, vanilla, or alcoholic beverages to increase the appeal of tobacco consumption [8, 9]. Flavor substances conceal the harshness of tobacco smoke [8], encourage the initiation and experimentation of tobacco use [10], decrease the perceived harm associated with smoking tobacco [11, 12], and contribute to the sustained use of tobacco [8]. Additionally, packaging elements such as colors, graphic representations of flavors, and advertising materials enhance the overall attractiveness of flavored tobacco products to encourage sustained tobacco consumption [12].

In the past, tobacco companies have used flavorings such as essential oils to enhance tobacco products [13]. Today, flavored tobacco products are further enhanced by technologies such as crushable capsules contained in the filter of the stick that release flavors when crushed. Crushable capsules were first introduced to the global stage in 2007 where their global market share has since increased from 0.23% in 2010 to 4.5% in 2020 [14]. In the Philippines, crushable capsules were introduced in 2013 [15] and have steadily increased their local market share from 2.5% in 2014 to 4.2% in 2018 [16]. Today, crushable capsules are the “fastest-growing segment of the combustible market” [17]. Together with innovations such as crushable capsules, “in-store marketing and display, colorful packs and nonconventional flavor names” are key drivers behind the consistent growth of the flavored tobacco market [18].

Young Filipino adults increasingly view novel innovations in flavored cigarettes, such as crushable capsules, as appealing [16]. Compared with those who use non-flavored tobacco products, consumers of flavored tobacco products are generally less likely to have cessation intentions [11, 19]. Additionally, consumers are likely to perceive flavored tobacco products as more favorable than non-flavored products [20]. For vulnerable groups such as women, the youth, and young adults, flavored tobacco products have a greater appeal and likelihood of use [21, 22]. Documents from the tobacco industry have shown that flavored tobacco products are intentionally designed as “starter products” meant to attract kids [23]. A recent study by the Institute for Global Tobacco Control revealed that within 200 m of schools, 90% of the 353 retailers surveyed sold flavored tobacco cigarettes [24]. As a result, the Philippines, with its significant youth population [25] and high smoking prevalence [1, 2], remains susceptible to adverse public health consequences from flavored tobacco use.

The Philippines has introduced and implemented several tobacco control policies to combat the country’s high smoking prevalence and health burden from tobacco use. However, the country has implemented little to no regulation of tobacco product flavors for cigarettes and cigars. In 2003, the national government enacted Republic Act No. 9211—or the Tobacco Regulation Act of 2003—to regulate the use, sale, distribution, and advertisements of tobacco products [26]. However, the act did not state any provisions to regulate or restrict flavored tobacco products [10]. In 2005, the Philippines became a signatory of the World Health Organization (WHO) Framework Convention on Tobacco Control (FCTC) [27]. The WHO FCTC emphasizes through Article 9 that there is no justification for using flavoring agents to enhance the attractiveness of tobacco products [8]. However, with no regulation to justify the prohibition of flavor additives in tobacco products, the market for flavored cigarettes continues to grow in the country. For example, the menthol cigarette market share in the Philippines slowly increased from 21.7% in 2014 to 22.4% in 2018 [16]. 

Several countries have already issued regulations to restrict flavored tobacco products. Existing flavor-related regulations emphasize the need to deter young people from tobacco use, prohibit specific flavors such as fruits, vanilla, and spices, and prohibit using flavor descriptors or flavor imagery on packaging [28]. Countries such as Brazil, Canada, Chile, Ethiopia, and Turkey have completely banned flavored cigarette sales [29]. In the US, Canada, the European Union (EU), Moldova, Turkey, and Mauritania, flavors are prohibited in parts of tobacco products, including paper and filters. The EU, Moldova, and Turkey specifically prohibit flavors in capsules [28]. In the EU, 27 EU Member States and the United Kingdom, under the European Tobacco Products Directive (TPD), banned characterizing flavors such as menthol, vanilla, or candy that conceal the smell of tobacco. In particular, the TPD banned flavors, such as menthol, that maintain over a 3% market share [30].

Existing literature has demonstrated the presence of support for policies regulating flavored tobacco products around the world. A study conducted in Brazil discovered that the majority of smokers supported the implementation of a ban on menthol and all additives [31]. In another study conducted in the U.S., 62.3% of adults endorsed a policy banning the sale of menthol cigarettes [32]. In comparison, 57.3% supported a policy prohibiting the sale of all tobacco products [32]. Multiple studies have confirmed an increase in quitting behaviors by menthol cigarette smokers after a restriction on menthol cigarette sales [33]. One related study revealed that menthol smokers, including young adults, support a ban on characterizing flavors and would attempt quitting if such a ban was enacted [34]. 

To date, few studies have examined the landscape of flavored tobacco products in the Philippines [10, 16]. The most recent data on the flavor profiles of the Philippine cigarette market date back to 2021 in a study conducted by Cohen and colleagues, which collected data on flavored cigarettes in the Philippines in 2016 [35]. This paper aims to describe the current landscape of both flavored cigarettes and cigars in the Philippines from the last seven years using previous data as a baseline. In particular, this paper will examine current flavor descriptors used on cigarette and cigar packs and other packaging features that characterize these flavor descriptors. Additionally, this paper outlines key tobacco company tactics incorporated in flavored cigarettes and cigars and what these implications entail for existing tobacco control regulations.

## Methods

### Design

This study primarily adapted the research design and protocol of the Tobacco Pack Surveillance System (TPackSS) and its 2015 Field Collection Protocol developed by the Institute for Global Tobacco Control (IGTC) at the Johns Hopkins Bloomberg School of Public Health [36]. 

We collected our samples from August 2023 to September 2023. Following the TPackSS protocol, our team collected unique cigarette and cigar packs from four (4) cities in each region of the Philippines (National Capital Region, Balanced Luzon, Visayas, and Mindanao) with the highest populations (Quezon City, Antipolo City, Cebu City, and Davao City respectively). We selected 12 neighborhoods (or *barangays*) for each city based on unique characteristics such as population density, economic significance, and geographic location. We used these characteristics as a proxy for socioeconomic classification, the primary criteria specified in the TPackSS protocol. We used proxy data in light of the absence of data on socioeconomic classification from local government units and publicly available databases. Within each neighborhood, our team sampled six (6) main vendor types, including *sari-sari* stores (or neighborhood stores), convenience stores, mall kiosks, street vendors, supermarkets, and department stores.

Our data collection procedure commenced with the walking protocol stipulated in the TPackSS protocol. Our team identified a commercial hub as the starting point for data collection in each neighborhood. Our team walked for five minutes from the commercial hub until we came across one of our six required vendor types. Our team purchased one of every unique pack available for sale. The TPackSS protocol defines unique packs as “any pack with a different design or feature, including packs differing in stick count, size, brand name presentation, colors, cellophane, and inclusion of a promotional item” [36]. In the succeeding neighborhood(s), our team purchased one of every unique pack available for sale that had not yet been purchased from the previous vendor(s).

After purchasing all possible products for the day, our team assigned a code to each pack, took pictures based on the guidelines specified by the TPackSS protocol, and segregated each pack into individual ziplock bags. Our team organized all the photos into Google Drive folders for systematic documentation.

### Coding

After collecting sample packs, our team coded each pack based on the TPackSS codebook [37]. The codebook accounts for several aspects of packaging, including structural elements such as size and shape and graphic elements such as brand or flavor descriptors. Two trained researchers conducted two rounds of coding to ensure the accuracy of the reported results. A third researcher participated in the second round of coding to resolve any discrepancies encountered in the first two sessions of individual coding from the first and second researchers.

### Sample

The inclusion criteria for this study include flavored cigarette and cigar packs, defined as those that feature flavor descriptors on the package or contain crushable capsule technology. Out of the 106 cigarette and cigar packs we collected, only 62 packs fit the inclusion criteria. Given that this study is an update of the 2021 study by Cohen and colleagues [35], the same exclusion criteria were followed: samples that did not have flavor descriptors, had flavor descriptors such as “original” or “full flavor”, or were not crushable capsule products. However, unlike the original study, we included tobacco flavor descriptors given the frequency of tobacco flavor descriptors we discovered.

### Analysis

We conducted a thematic analysis to characterize the flavor profile of all 62 packs. A combination of deductive and inductive analysis was employed to identify these themes. We identified five categories, namely, menthol, concept descriptors, beverages, tobacco, and other flavors. Menthol includes descriptors that explicitly use the terms “menthol” or “mint”. According to Cohen and colleagues, concept descriptors are “terms that imply that some type of flavor, sensation, taste, or aroma awaits the consumer” [35]. Beverage flavor descriptors reference beverages such as alcoholic beverages or coffee. Tobacco flavor descriptors include descriptors that explicitly use “tobacco” and other words or phrases describing the flavor of tobacco. Other flavor descriptors include those that reference sweet or floral flavors.

## Results

### Sample breakdown

Of both cigarettes and cigars, 59 (55.66%) out of 106 had flavor descriptors. 49 (83.05%) were cigarettes, while 10 (16.95%) are cigars (see Table 1). Of the 106 samples, 86 (81.13%) were non-crushable capsule products, while 20 (18.87%) are crushable capsule products. 10 packs (45%) with concept descriptors are crushable capsule products. Three out of 20 (15%) crushable capsule products did not feature a flavor descriptor but were included in the graphic analysis of the study. This resulted in 62 samples in total.

**Table 1:** General overview of samples

Category	Subcategory	Count	Percentage
Total Sample	Cigarettes	91	85.85%
	Cigars	15	14.15%
	Total	106	100.00%
All products	With flavor descriptors	59	55.66%
	Without flavor descriptors	47	44.34%
	Total	106	100.00%
Net Sample	Cigarettes	49	83.05%
	Cigars	10	16.95%
	Total	59	100.00%
Capsule products	Non-capsule	86	81.13%
	Capsule	20	18.87%
	Total	106	100.00%

As reflected in Table 2, the majority of flavored packs originated from Philip Morris Philippines Manufacturing Inc. (PMFTC) (30.51%), followed by Japan Tobacco International (JTI) Asia Manufacturing Corporation (25.42%). 13 packs (22.03%) did not specify the manufacturer on the package. Without such information, our team conducted further research to identify the manufacturer. Unspecified manufacturers include Tabaqueria de Filipinas (Philippines), Manifatture Sigaro Toscano SpA (Italy), and Tabacalera Incorporada (Philippines). The three crushable capsule products with no flavor descriptors included in our study were obtained from JT International Asia Manufacturing Corp. and KT&G. Some of our sample packs can be considered illicit products or are being sold illegally, as evidenced by the lack of Bureau of Internal Revenue (BIR) tax stamps. Only one of these illicit packs specified a manufacturer on the packaging (SLH International Management S.A.), while the rest of the illicit packs did not specify any manufacturer. Our team did not trace the remaining illicit packs to a manufacturer.

**Table 2:** Manufacturer distribution of cigar and cigarette product samples with flavor descriptors

Manufacturer	# of Packs	Percentage	Country of Origin
PMFTC, Inc.	18	30.51%	Philippines, joint venture
JT International Asia Manufacturing Corp.	15	25.42%	Philippines, subsidiary
Not specified	13	22.03%	
KT&G	5	8.47%	Korea
La Suerte Cigar and Cigarette Factory	2	3.39%	Philippines
China Tobacco Zhejiang Industrial Co., Ltd.	2	3.39%	China
SLH International Management S.A.	1	1.69%	Switzerland
Gold Tree Tobacco Manufacturing Corp.	1	1.69%	Philippines
British American Tobacco Group	1	1.69%	United Kingdom
Anglo American Tobacco Corporation	1	1.69%	Philippines
Total	59	100.00%	

Table 3 outlines the complete profile of flavor descriptors in our sample. The team identified flavor descriptors based on explicit wording on sample packs that connote flavors or flavor sensations. After conducting a thematic analysis, our team identified five categories of flavor descriptors: menthol (40.58%), concept descriptors (26.09%), tobacco (18.84%), beverages (7.25%), and other flavors (7.25%). Out of the 59 packs with flavor descriptors, 10 packs (16.95%) had flavor descriptors that fit more than one category. Given this, the team identified a total of 69 flavor descriptors.

**Table 3:** Flavor Profile of flavored cigar and cigarette product samples

Category	Count	Percentage	Subcategory	Examples
Menthol	28	40.58%	Menthol Mint	Menthol, black menthol, menthol kings, menthol release, menthol boost, crush release menthol Mint burst max, cool mint, extreme mint, mint splash
Concept descriptor	18	26.09%		Max cool, activate max, mint burst max, premium blend, aroma blend, ice blast mega, summer zest, remix cool, remix summer, American blend, crafted ice, crafted red, fusion purple, ice wave, best toasted American blend, aroma blend
Tobacco	13	18.84%	Regional Type	Virginia type, Virginia tobaccos American blend, Cuban seed tobacco Extra tobacco, rested tobaccos, 100% tobacco
Beverage	5	7.25%	Alcohol Coffee	Mojito ball, Mojito capsule, red wine, mojito double Rosso caffè
Other flavors	5	7.25%	Sweet Floral	Vanilla creme, vanilla, mild & sweet, honey nectar Cherry blossom
Total	69	100.00%		

For crushable capsule products, we found four categories of flavor descriptors: concept descriptors (44%), menthol (28%), beverages (16%), and unknown (12%).

### Flavor descriptors

Menthol flavor descriptors comprised the majority of flavor descriptor categories in all sample packs. They are divided into two subcategories: menthol and mint. Flavor descriptors from the menthol subcategory explicitly use the word “menthol” and can include variations such as “menthol release”, “black menthol”, and “menthol boost”. Moreover, descriptors in the subcategory explicitly use the word “mint” and include examples such as “mint splash”, “mint burst max”, and “extreme mint”.

The share of concept descriptors in our five categories closely followed that of the menthol flavor descriptors. Concept descriptors are commonly observed among crushable capsule products. “Fusion purple”, “summer zest”, and “activate max” are examples of concept descriptors from our samples.

Tobacco flavor descriptors are those that make explicit use of regional or type references to tobacco. Examples of regional flavor descriptors include “Virginia tobaccos American blend” and “Cuban seed tobacco”. Flavor descriptors that describe the type of tobacco include “Extra tobacco”, “rested tobaccos”, or “100% tobacco”.

Beverage flavor descriptors explicitly reference alcoholic beverages or coffee. Examples of flavor descriptors from the alcoholic beverage subcategory include “red wine” or “mojito double”. Our team only discovered one flavor descriptor in the coffee subcategory: “Rosso caffè”.

Sample packs with other flavor descriptors featured flavors in the sweet and floral subcategories. Sweet flavor descriptors include “vanilla creme”, “honey nectar”, and “mild & sweet”. One floral flavor descriptor, “cherry blossom”, was observed.

Among the 59 samples with flavor descriptors, 20 (18.87%) were crushable capsule products (see Table 4). Concept descriptors such as “fusion purple”, “activate max”, and “remix cool” make up the majority (40%) of flavor descriptors on crushable capsule products. Menthol closely follows concept descriptors comprising 32% of all crushable capsule products. Beverage flavor descriptors such as “mojito” comprise 16%. Three sample packs (12%) were identified as crushable capsule products but did not indicate any flavor descriptor on the packaging.

**Table 4:** Flavor descriptors on crushable Capsule products

Category	Count	Percentage	Examples
Concept descriptors	11	44.00%	Max cool, activate max, ice blast mega, menthol, ice blast mega, remix cool, remix summer, max cool, ice wave, menthol boost
Menthol	7	28.00%	Menthol, mint burst max, cool mint, menthol boost
Beverages	4	16.00%	Mojito capsule
Unknown	3	12.00%	
Total	25	100.00%	

A few flavor descriptors fit more than one category. For example, we identified “mint burst max” as both a menthol flavor and concept descriptor. Other packs also feature more than one flavor descriptor. In one instance, a sample pack indicated “summer zest” on the front of the pack but indicated “20 menthol cigarettes” on the side of the pack. Flavors such as “original” or “full flavor” were not counted as flavor descriptors.

### Graphic characteristics and other appeals

**Fig. 1:**
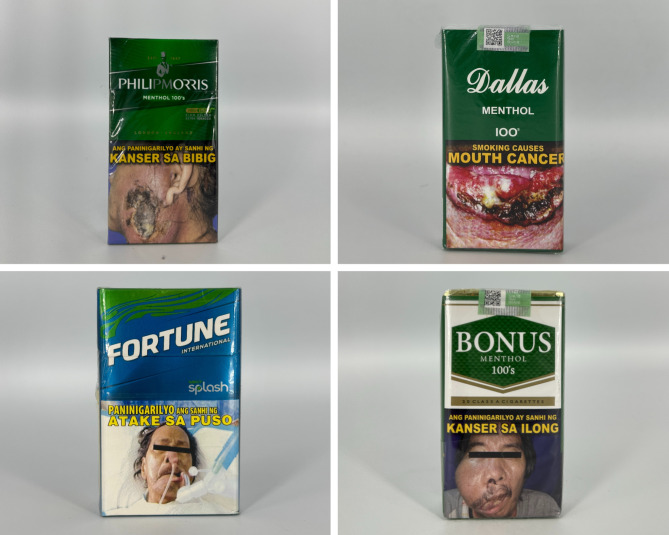
Packs with menthol flavor descriptors

Packs with menthol flavor descriptors often feature green, blue, or black as the primary or accent color(s) (see Fig. 1). Gold accents and ombre or gradient colors connote a sense of luxury and enhance flavor descriptors such as “summer zest”. Text styles such as serif or script create premium appearances. When promoting a futuristic appeal, reflective borders, rugged typefaces, three-dimensional logos, and graphics such as flaresare common. In terms of graphic elements, stylistic lines, patterns, and textures are commonly used. For example, textures depict certain images such as a fingerprint or the brand logo, and also accentuate certain parts of the pack in the corners or the borders. Additionally, the V-cut shape is commonly featured on packs to frame the brand logo. Our team observed images such as a port or mountains depicted on the principal display areas of some packs.

**Fig. 2:**
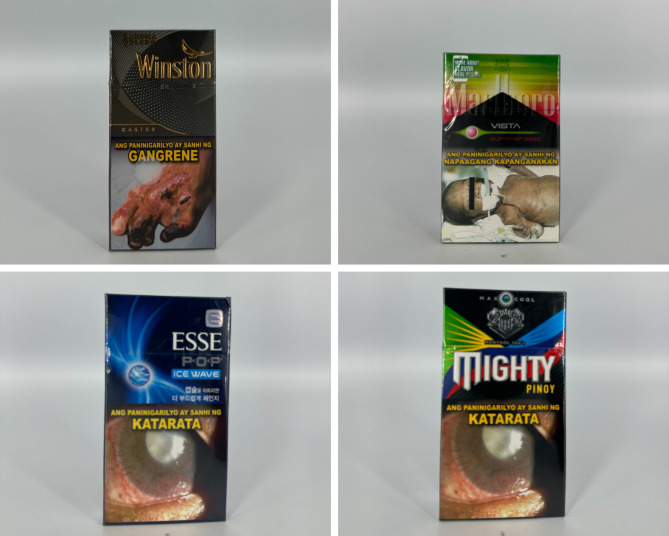
Packs with concept descriptors

**Fig. 3:**
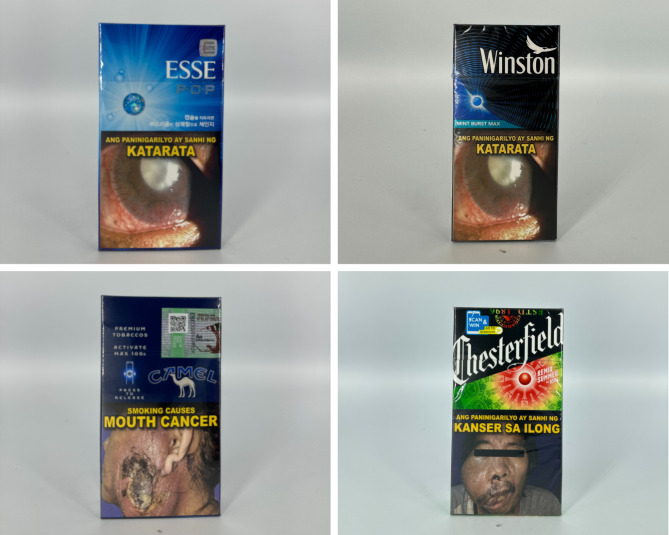
Crushable capsule products

For concept descriptor packs (see Fig. 2), graphic imagery associated with the crushable capsule feature is common (see Fig. 3). Ball shapes and other features such as ripples are used to depict the crushable capsule of the product. In one of the crushable capsule packs, there is an image of a crushable capsule in a cigarette filter with arrows pointing toward the capsule, accompanied by the phrase “press to release”. This indicates that imagery is used as a form of instruction for users. The use of ombres and linear or radial gradients is also common in the majority of concept descriptor packs. Black is the most common primary color, with blue, green, purple, and gold as common accent colors. Pack colors are also used to correspond to flavor descriptors, one example being “Crafted Red”, which has a corresponding red V-cut shape on the top of the box. Reflective or holographic textures and designs connote futuristic appearances .

**Fig. 4:**
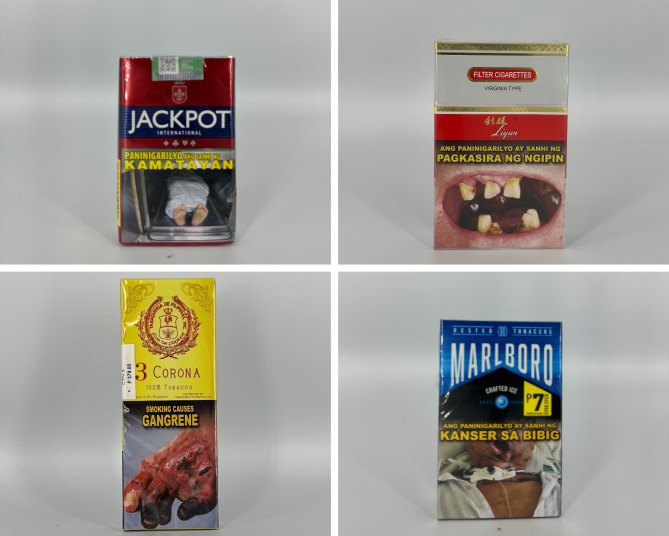
Packs with tobacco flavor descriptors

The primary colors of tobacco flavor descriptor packages vary (see Fig. 4). Green, red, black, white, and yellow, are common primary colors with gold as a common accent color. Few packs had icon illustrations of tobacco leaves, with others having imagery such as a port and a gold American eagle. Other accents, such as a signature, linear textures, and stylistic lines, are commonly used.

**Fig. 5:**
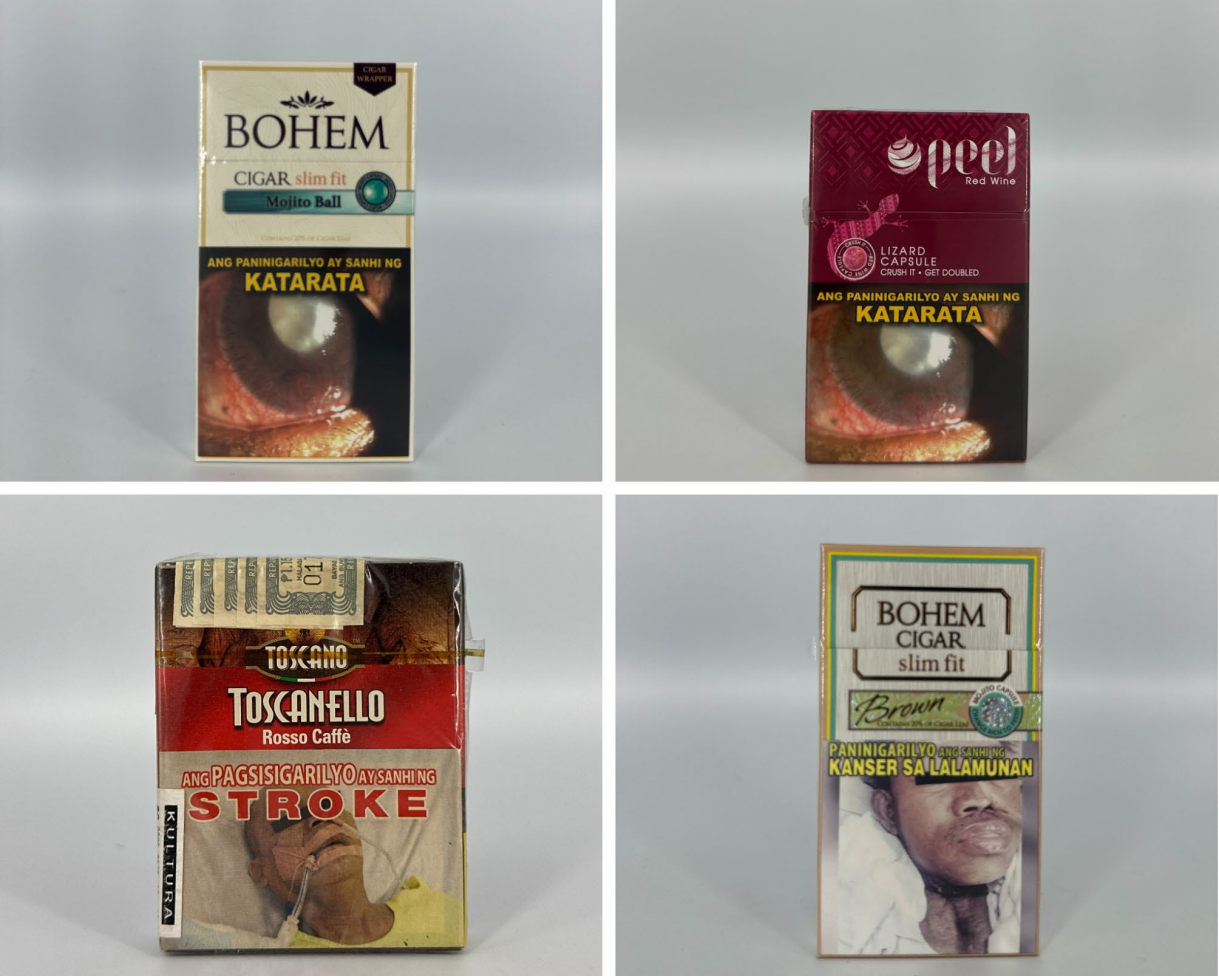
Packs with beverage flavor descriptors

Beverage packs often feature more creative and premium designs (see Fig. 5). Reflective or holographic accents, engraved text, and three-dimensional textures are commonly used. These packs also use more explicit images, including a lizard, a map of Cuba, and tobacco leaves. The colors are used toconnect with the flavor descriptor. One pack flavored “red wine” uses a red wine color as the primary pack color. Colorful borders, patterns using diamond shapes, and textural lines are commonly used .

**Fig. 6:**
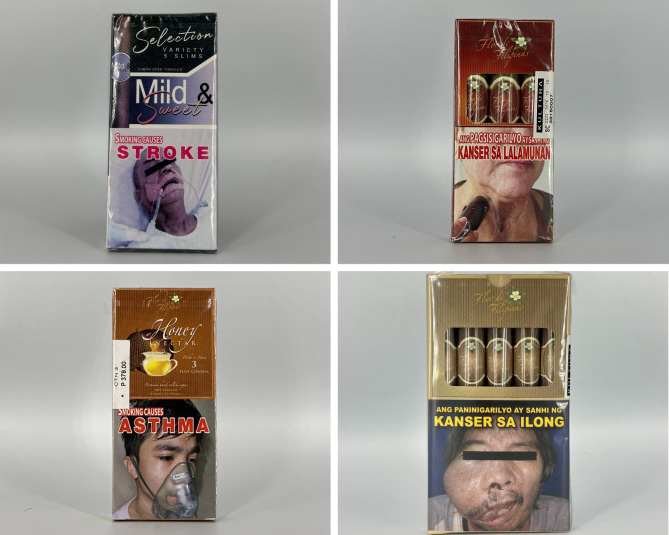
Packs with other flavor descriptors

The majority of packs with other flavor descriptors have open packaging that allows consumers to view the product inside (see Fig. 6). Images such as a cherry blossom, a jar of honey surrounded by bees, or milk with flowers and vanilla beans are common. Moreover, the colors are slightly duller and muted than those of packs from other categories. Primary pack colors include maroon, brown, or white with reflective gold outlines and accents.

In addition to their graphic characteristics, our team observed that flavor descriptors on crushable capsule products are often accompanied by other taglines that emphasize both the experience and sensation that comes with using the product. Examples of these phrases include “A new refreshing summer taste experience”, “Pop it fresh, feel the CHANGE” and “The distinctive scent of Mojito Double will take you to the exotic scenery of Cuba”.

## Discussion

This study is an update on 2016 data from the Philippines based on the methodology used in the study by Cohen and colleagues (2021) which examined flavored cigarettes in low- to middle-income countries, specifically Bangladesh, Brazil, China, Indonesia, Philippines, Russia, Thailand, and Vietnam [35]. 

The results from both studies presented several similarities. First, menthol and concept descriptors comprise the top two shares of flavor descriptors in both studies. In the study by Cohen and colleagues, menthol took the leading share of their flavor descriptor categories in all countries except India and Russia [35]. In the current study, menthol cigarettes also comprised the leading share of the samples collected at 40.58%. Both studies highlight the continued dominance of menthol in the flavor marketplace in low- to middle-income countries.

On the other hand, there are five main differences between both studies. First, while the study by Cohen and colleagues (2021) did not include tobacco flavor descriptors [35], this study included tobacco flavor descriptors. Given the frequency of tobacco flavor descriptors in our sample, we decided it was best to feature these descriptors in our study to provide a better understanding of how tobacco flavored are described on these packs.

Second, while menthol comprised the majority share of flavor descriptors for crushable capsule products in the study by Cohen and colleagues (2021) [35], we discovered that concept descriptors took the majority share of our crushable capsule products. Nonetheless, for both studies, both menthol and concept descriptors occupied the top two total shares of flavor descriptors for crushable capsule products.

Third, while the leading share of flavors in India and Russia was fruit or citrus, we did not discover any fruit or citrus flavors in our study. Fourth, this study examined flavored cigars while the study by Cohen and colleagues (2021) did not include cigars in their sample [35]. We discovered that some cigar samples are incorporated with crushable capsule technology and that most of their flavor descriptors fall under our “other” category (specifically sweet and floral flavors).

Fifth, there is a difference in terms of manufacturer’s market share in flavored tobacco products between 2016 and 2023. In the study by Cohen and colleagues (2021), KT&G and Philip Morris Fortune Manufacturing Corp. (PMFTC) command the leading shares in their samples of flavored tobacco products from low- and middle-income countries [35]. Meanwhile, in the samples we collected in 2023, PMFTC and Japan Tobacco International commanded the leading shares in our sample of flavored tobacco products. KT&G ranked fourth in our sample while unspecified manufacturers take up the third ranking in our products. Such a difference shows that the monopoly of cigarettes and cigars has changed considerably in the last 7 years.

Notably, out of our samples’ nine manufacturers, a third of these are local, with two transnational companies in the top two shares of flavored products. This points to the trend that flavor concepts are more common among products from transnational manufacturers, specifically Philip Morris Fortune Tobacco Corporation (PMFTC) and Japan Tobacco International. There is a similar trend in crushable capsule products. These findings support the Southeast Asian Tobacco Control Alliance’s (SEATCA) evidence that PMFTC maintains a 70.5% market share in the Philippines with Japan Tobacco International following behind at 26.5% market share [38]. Our findings also corroborate Euromonitor International data which shows that Philip Morris Fortune Tobacco Corporation (70.7% market share) and Japan Tobacco International (29%) dominate the cigarette market [39].

### The influence of flavor descriptors and flavor imagery

When comparing the 2021 study by Cohen and colleagues and the results of this study, we found that menthol and concept descriptors maintained the top two shares of flavor descriptor categories in both studies. Notably, both studies show that menthol continues to dominate the flavor marketplace in low- and middle-income countries. Recent research shows that the market share of menthol and flavored tobacco products increased from 2005 to 2019 in low- and middle-income countries, especially in Nigeria, Russia, Guatemala, Peru, Kenya and Zambia [15]. This was driven by tobacco company marketing strategies which included point-of-sale advertising and promotion, package design, flavor descriptors, and marketing menthol and flavored products as less harmful [15]. Tobacco companies have a history of targeting women with menthol-flavored brands, and using product design through added flavors, flavor capsules and dials to control menthol delivery in Southeast Asia [40].

Existing literature suggests that flavors and flavor packaging can significantly influence the perception, initiation, progression, and continuation of tobacco product use [8, 10, 11]. In response to increasingly stringent restrictions on tobacco advertising, promotions, and sales, tobacco companies have turned to other novel marketing tactics such as flavor descriptors or flavor imagery, to appeal to target audiences [41]. A study in Brazil compared flavored packs with descriptors and the same packs without the descriptors [42]. They found that flavored plain packs with flavor descriptors were rated higher in terms of brand appeal, smoothness, and taste compared to the same packs with no descriptors [42].

Our study revealed that flavor descriptors are complemented or enhanced by connoting sensations and experiences, creating a sense of premium versus non-premium flavors, and establishing references to other health-harmful products such as alcoholic beverages. These findings suggest that tobacco companies strategically target certain demographics or lifestyle aspirations to appeal to and to connect with diverse consumers. This supports Lewis and Wackowski’s argument: Flavored cigarettes are designed as innovations that cater to product preferences and reach target audiences to increase market share [43].

The ubiquity of certain flavors such as menthol can also be a means to appeal to popular consumer preferences. Young Filipino adults will likely perceive certain flavors such as menthol as less harmful than other flavored cigarettes [16] because menthol masks the harshness of cigarette smoke by numbing the throat with a cooling sensation [44]. Menthol provides consumers with a false sense of flavored cigarettes being “safer”, which is further fuelled by tobacco companies marketing menthol cigarettes as such [45]. However, the literature suggests that menthol cigarettes are not necessarily safer than other cigarette flavors [46 −52].

For example, menthol cigarette smokers are likely to be more heavily dependent on nicotine,[46 −48] which can consequently reinforce smoking behaviors [42]. The use of menthol cigarettes has also been found to increase the number of children who initiate and experiment with smoking, increase the addictive potential of smoking among youth, and increase the risk of relapse[49 −52]. Given that the youth in the Philippines have alarmingly high smoking prevalence rates [2], menthol cigarettes raise significant public health concerns in the country. Given that the Philippines has one of the highest market shares of menthol cigarettes in the world [17], menthol cigarettes are a critical public health challenge.

Flavor imagery also has a significant role in flavored tobacco product packaging. One study found that flavor imagery can establish consumer recognition for flavor technologies such as crushable capsules [47]. The same study found that colors can signify flavors and increase the attractiveness of the pack. Brand, pack, and technology recognition can serve as potential gateways or contributors for the initiation and continued use of flavored tobacco products [35]. This raises concern when contextualized in the 2021 study by Cohen and colleagues which found that the Philippines had the highest proportion of unique packs with flavor descriptors and/or imagery [35]. With the high ubiquity of both flavor imagery and flavor descriptors in packs from the Philippines, the demand for flavored tobacco product regulation is critical for protecting public health.

### Innovations and technologies in tobacco products

The use of menthol cigarettes, coupled with technologies such as capsule technology, can increase the sales of cigarettes [43]. Unfortunately, parallel to the country’s high market share of menthol cigarettes [16], the Philippines has a similarly high share of crushable capsule cigarettes [18]. Crushable capsule cigarettes account for more than 30% of cigarettes in a few countries [41]. They are perceived as having more pleasant sensations on the throat, better tasting, and more fun smoking [53]. 

Our findings suggest that crushable capsule cigarettes offer more than pleasant sensations; they also offer experiences through the frequent use of concept descriptors. Inviting taglines that enshrine “refreshing” and “exotic” experiences during smoking complement concept descriptors. These novel marketing tactics have the potential to dramatically increase the appeal of capsule cigarettes, especially among younger consumers. One study revealed that young adults who smoke identified the youth as the target audience for flavor capsule cigarettes [54]. Other studies confirmed that capsule cigarettes are viewed as attractive by young adults [16] and can positively contribute to young women’s smoking experiences by allowing them to personalize their smoking experiences [55]. Crushable capsules, and other innovations in tobacco products, must be regulated by more robust legal compliance and regulatory requirements to prevent higher rates of tobacco smoking among intended audiences.

Meanwhile, data from Euromonitor on cigars and cigarillos in the Philippines show that the sale of cigars has increased from Php 398.6 million (USD $6.893 million) in 2017 to Php 577.1 million (USD $9.980 million) in 2022 [39]. This is but a small market compared to cigarette sales which were estimated to have increased from Php 216.6 billion ($USD 3.747 billion) in 2017 to Php 418.1 billion (USD $7.232 billion) in 2022 [39].

Our samples show us that a higher proportion of cigars are flavored compared to cigarettes and that cigars incorporate innovations such as unconventional physical structures and capsule technology. For example, we have cigar samples that had the structural characteristics of a cigarette but used a leaf as its wrapping. These cigars also included crushable capsules. Cigars are evolving into products that could be more appealing to a wider range of consumers, which calls for further investigation into the cigar market and its use patterns.

### Opportunities for regulating flavored tobacco products

Contextualizing the Philippines within the global landscape of flavored tobacco regulation will provide significant room for supporting the rationale behind proposed legislation on flavor bans and their potential effectiveness for protecting public health.

The lack of regulation of flavors, ingredients, or additives [27] runs contrary to the commitment of the Philippines as a party to the WHO FCTC, which prohibits the use of flavors or flavor additives that can increase the appeal of tobacco products [8]. As such, policymakers should consider regulating flavors and banning flavor descriptors to align with the WHO FCTC and its guidelines to prevent tobacco use and protect vulnerable groups such as the youth and women.

The foremost tobacco control intervention to implement is monitoring the market, perceptions, and demographics targeted by flavored tobacco products stipulated by the WHO FCTC and the M in the MPOWER package [53]. This intervention can inform efforts to reduce the sales of flavored tobacco products. Effectively reducing the sales of flavored tobacco products must be implemented through countrywide comprehensive approaches instead of partial restrictions [56].

Policymakers can consider enforcing a comprehensive ban on flavors. A flavor ban can curb the use of cigarettes by youth and young adults [34]. A related study examining the effectiveness of the U.S. Food and Drug Administration’s national ban on flavored cigarette products discovered that the ban was significantly associated with reduced cigarette use among youth and young adults [57]. However, such a ban must be supported by stringent enforcement [58] and comprehensive policies that prevent tobacco companies from exploiting policy loopholes [59].

For example, a ban on flavors can be complemented by a ban on flavor descriptors and flavor imagery [60]. Such regulations will counteract the historical efforts of tobacco companies to circumvent tobacco flavor policies by removing explicit flavor descriptors while retaining flavor chemicals [60]. A ban on flavors and flavor descriptors is also crucial to combat their complementary effect in boosting the appeal and uptake of tobacco products [12]. Likewise, it is worth considering banning flavors occupying a certain level of market share, inspired by the European TPD’s ban on flavors with more than a 3% market share [30]. Although restricting flavors may not eradicate flavored tobacco product use, it may substantially reduce its prevalence [34].

Policymakers should also consider plain packaging as an intervention to disrupt the flavor market. A study in Brazil discovered that removing flavor imagery and descriptors through plain packaging significantly reduced the taste ratings of flavored brands [61]. A recent study found that Filipinos recognized the potential of plain packaging to “make health warnings on tobacco products more effective, reduce their attractiveness, eliminate misleading information, and increase the noticeability and effectiveness of health warnings” [62]. While tobacco companies have opposed plain packaging measures, Thailand and Singapore have already implemented plain packaging with success amid opposition from tobacco companies [63].

## Conclusion & recommendations

This study updates and extends the available evidence on flavored tobacco products in the Philippines by providing a snapshot of the flavored tobacco product landscape from 2016 to 2023. Our study revealed five main categories of flavor descriptors among our sample of 62 flavored cigarette and cigar products: menthol, concept descriptors, tobacco, beverages, and other flavors. Menthol and concept descriptors maintain their dominant share in the market for both non-crushable capsule and crushable capsule products. Crushable capsule products mostly feature concept descriptors coupled with phrases that emphasize the experience or sensation associated with the product’s technology. Menthol and concept descriptors are more common for cigarettes while sweet and floral flavors are more common for cigars. Meanwhile, a higher proportion of cigars are flavored compared to cigarettes, which calls for further investigation into their market share and use patterns. While our study corroborates the initial findings of the study by Cohen and colleagues (2021), we offer new information on flavors for other types of tobacco products. Moreover, this study provides additional value by examining flavored cigars, which were not included by Cohen and colleagues (2021). We reiterate the recommendation to regulate flavored tobacco products in terms of flavor substances, flavor descriptors, and flavor imagery to reduce their appeal and curb smoking prevalence and rates of tobacco-induced diseases. Policymakers should collaborate with public health advocates, ensure the successful establishment and implementation of monitoring systems, introduce flavored tobacco product bans, and push for plain packaging to disrupt the flavor market and protect public health.

## Data Availability

No datasets were generated or analysed during the current study.

## Declarations

### Authors’ information

SA and PC are research assistants for the Tobacco Control and Governance (TCG) Program of the Ateneo Policy Center (APC), School of Government (SOG), ADMU. AS program manages TCG while GA is an appointed non-resident research fellow of SOG.
